# A Review of Preparation Methods, Mechanisms, and Applications of Bio-Based Phenolic Nanoparticles

**DOI:** 10.3390/foods15172970

**Published:** 2026-08-24

**Authors:** Yulu Ma, Wenqi Deng, Menghuan Sun, Xiaojing Jiang, Jingyang Hong

**Affiliations:** College of Smart Agriculture (Research Institute), Xinjiang University, Urumqi 830046, China

**Keywords:** polyphenol nanoparticles, preparation method, mechanism of action, application

## Abstract

With the improvement in people’s quality of life, issues related to food health and nutritional supplementation have attracted widespread attention. Polyphenols have been established as bioactive compounds that exert multiple beneficial effects on human health. However, due to their chemical instability and susceptibility to environmental factors, polyphenols face restrictions during food processing and exhibit decreased absorption rates in the human body. This review critically investigates the encapsulation of polyphenols via nanoparticle technology, with emphasis on preparation methodologies, mechanistic pathways, and application performance, particularly with respect to oxidative stability in oil-based systems. The present review provides a comprehensive discussion on the influence of nanoparticle encapsulation on the bioaccessibility of polyphenols, with the goal of enhancing their bioaccessibility and broadening their applicability across the food, cosmetic, and pharmaceutical sectors. These findings offer significant reference value for the future incorporation of polyphenols into food matrices.

## 1. Introduction

Polyphenols are recognized as crucial secondary metabolites abundantly distributed in plant-based foods, including fruits, cereals, and vegetables, and are conventionally classified into flavonoids and non-flavonoids [1]. These compounds exhibit antioxidant, anticancer, gastrointestinal regulation, and hypoglycemic activities, thereby exerting promising health-promoting effects on humans. However, certain polyphenolic compounds suffer from poor solubility and limited stability, and others demonstrate low digestive and absorptive efficiency in the human gastrointestinal tract, which collectively constrains their application in food processing.

Currently, nanoparticle technology has been increasingly employed to improve the stability, bioavailability, and oral uptake of polyphenolic compounds. Nanoparticles, typically ranging from 10 to 1000 nm in diameter, serve as delivery carriers; smaller-sized nanoparticles are capable of effectively penetrating cell walls and manifest high structural stability. Furthermore, nanoparticle technology can modulate the migration rate, diffusion capacity, thermal stability, and specific surface area of particles, consequently regulating the bioavailability of low-molecular-weight substances [2].

Polyphenol-based nanoparticles, owing to their intrinsic antioxidant and antimicrobial properties, have emerged as a promising platform for food preservation applications. The incorporation of these nanoparticles into oil systems not only improves the bioavailability of polyphenols but also potentiates their biological activities. The present work offers novel perspectives on the potential utilization of polyphenols. This review critically summarizes the preparation strategies and underlying mechanisms of polyphenol nanoparticles, along with their applications in the food, pharmaceutical, and cosmetic sectors, and delineates the theoretical and practical significance of the available literature.

The relevant literature was identified through systematic searches of the Web of Science, Scopus, and PubMed databases using keywords such as ‘polyphenol nanoparticles’, ‘protein-polyphenol’, ‘polysaccharide-polyphenol’, ‘lipid nanocarriers’, and ‘encapsulation’. Additional references were obtained by manually screening the bibliographies of retrieved articles. We included peer-reviewed original research and review articles published in English that addressed the preparation, interactions, stability, or applications of bio-based polyphenol nanocarriers. Studies focusing exclusively on synthetic polymer carriers or non-food applications were excluded.

## 2. Classification of Polyphenol Nanocarriers by Matrix Material

Nanodelivery systems have demonstrated considerable potential in addressing the poor bioavailability of polyphenols, which are otherwise constrained by low aqueous solubility, chemical instability, and inadequate intestinal permeability. Following oral administration, nanoparticle-encapsulated polyphenols are primarily absorbed via endocytosis, passive transcellular transport, and paracellular routes [3]. Notably, nanoparticles with a diameter of less than 500 nm are not removed by the macrophage system, thereby prolonging the circulation time of the encapsulated bioactive compounds in vivo [4].

**Figure 1:**
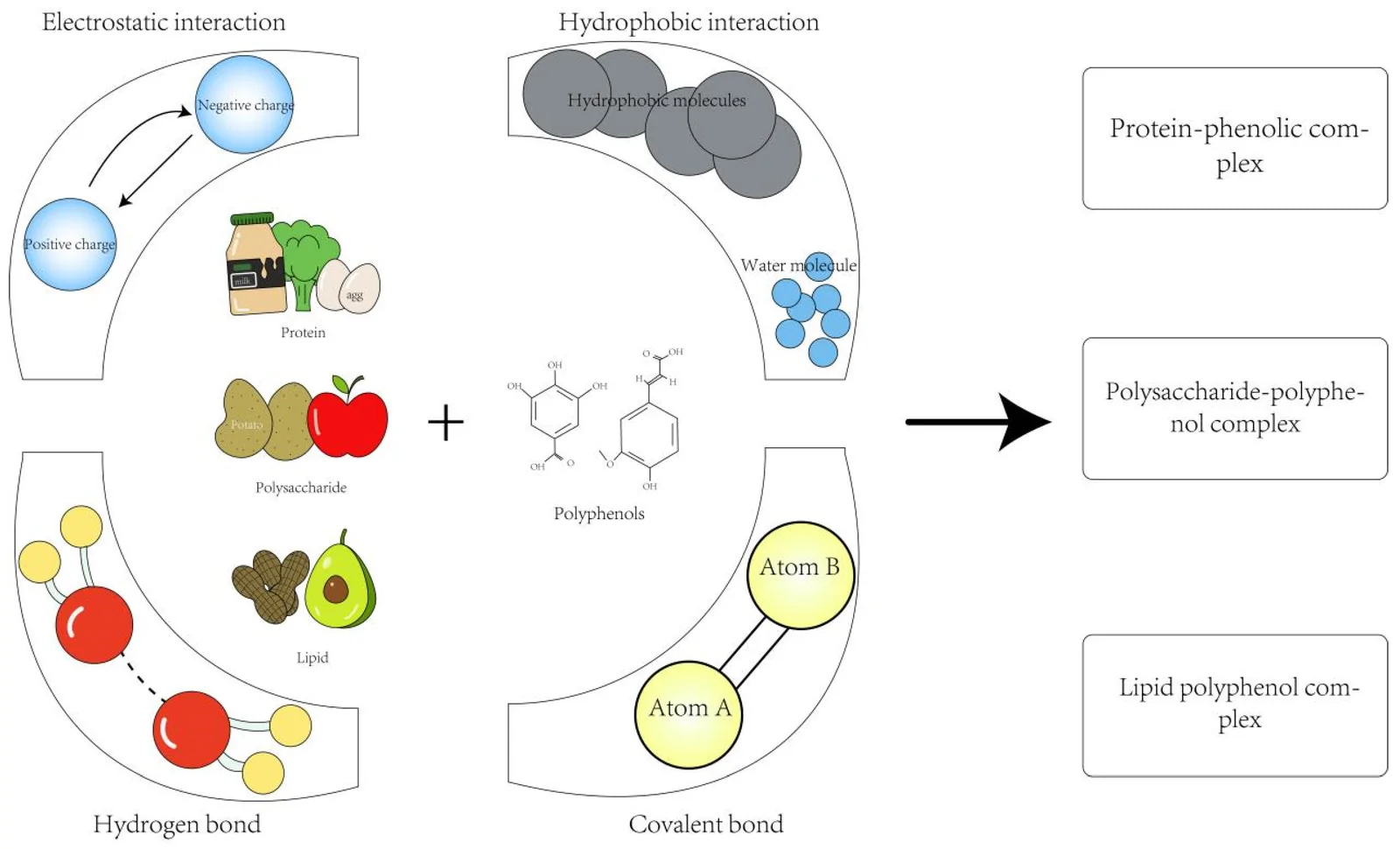
Schematic illustration of molecular interactions and hierarchical assembly in bio-based polyphenol complex formation.

According to the origin of the matrix materials, conventional delivery systems can be broadly categorized into two major classes: biopolymer-based nanocarriers and synthetic polymer-based nanocarriers. In comparison with traditional synthetic carriers, bio-based nanocarriers are distinguished by their small dimensions (typically below 100 nm), high specific surface areas, and favorable biocompatibility, which collectively promote the efficient absorption and controlled release of polyphenols [5,6]. The molecular interactions that govern the assembly of these biopolymer-based carriers with polyphenols are illustrated in Figure 1, and the distinct characteristics of each carrier type are elaborated in Section 2.1, Section 2.2 and Section 2.3. The prospective advantages of this technology encompass enhanced drug targeting efficacy and diminished adverse effects [7]. Notably, substantial progress has also been achieved in the domains of food preservation and nutrient delivery [8].

### 2.1. Protein–Polyphenol Interactions

Proteins derived from plant and animal sources serve as the principal dietary reservoirs of amino acids and biofunctional constituents in foods. These bioactive entities have been increasingly recognized for their multifaceted physiological roles, encompassing weight regulation, hypotensive effects, enhanced thermogenesis and satiety, alongside favorable modulations of cardiovascular metabolic biomarkers [9]. Proteins and polyphenols not only serve as reservoirs of bioactive substances that are beneficial to human health and nutrient absorption, but their physicochemical interactions also critically influence food quality and stability [10]. One strategy to expand the functional applications of proteins involves assembling them into functional complexes or particulate structures [11]. Proteins and polyphenols are capable of forming complexes via both non-covalent interactions and covalent conjugation [12]. The molecular interactions between polyphenols and biopolymer matrices have been characterized using a variety of analytical techniques. Spectroscopic methods, including Fourier-transform infrared (FTIR) spectroscopy and fluorescence spectroscopy, are routinely employed to detect hydrogen bonding and polyphenol-induced conformational changes in protein hydrogen bonding and conformational changes in proteins upon polyphenol binding [13,14]. Zeta potential measurements provide evidence of electrostatic interactions by monitoring surface charge alterations [15]. Specifically, the phenolic hydroxyl groups (-OH) and catechol moieties of polyphenols can serve as hydrogen donors or acceptors, forming hydrogen bonds with the peptide carbonyl groups (-C=O) and amino side chains (-NH_2_, -COOH) of proteins [13,16]. Covalent conjugation between proteins and polyphenols may proceed via multiple mechanisms, including oxidative coupling and enzymatic crosslinking, depending on the specific phenolic and protein species involved [17,18]. These particles can serve as functional ingredients to improve food functionality and deliver polyphenol-associated health benefits [19]. For instance, composite nanofiber films based on corn protein/gelatin-proanthocyanidin-zinc oxide nanoparticles have been developed to prolong the shelf life of sweet cherries [20]. Protein molecules are typically composed of amino acid residues, and the diversity of their residue composition and sequential arrangement underlies the distinct identity of different proteins. The interaction between proteins and phenolic compounds is governed by the specific types of both proteins and phenolic compounds involved [13]. Such interactions can be directed toward hydrophobic or hydrophilic binding, depending on the available binding sites on the protein. Certain proteins, such as zein, exhibit a strong affinity for hydrophobic molecules and can therefore be utilized to fabricate nanoparticles for hydrophobic cargo [21]. Polyphenolic substances can be protected by protein carriers and retain their functionality prior to gastrointestinal transit, where they undergo decomposition before digestion—a phenomenon that has been confirmed for tea catechins in combination with animal- and plant-derived proteins [16]. Some protein–polyphenol particles are formed by adsorbing moderately polar polyphenolic substances onto proteins under pH conditions near the isoelectric point of the interacting protein, followed by mixing. The resulting particles are primarily held together via non-covalent bonds and are reversible upon post-digestion processing [22,23]. A recent clinical study concluded that, when polyphenols are consumed in the form of protein-rich legumes or dairy beverages rather than as capsules, no significant differences are observed in their bioavailability or in the excretory volume of their phenolic metabolites [24]. To date, a wide variety of protein–polyphenol complexes have been fabricated from diverse protein sources and phenolic compounds, demonstrating a broad spectrum of functional enhancements. Table 1 summarizes five representative systems that have been selected to encompass the major interaction types discussed above: covalent glycosylation (pea protein–curcumin), covalent conjugation (soy protein–catechin), non-covalent electrostatic/hydrophobic assembly (soy protein fiber–EGCG/curcumin), and non-covalent association (plant protein–apple polyphenols; serum albumin–catechin). For instance, pea protein isolate has been reported to form covalent complexes with curcumin, improving emulsification and controlled release properties [17]; soy protein isolate binds covalently with catechin to enhance its bioavailability [18]; and non-covalent interactions between soy protein fiber and polyphenols, including curcumin and epigallocatechin gallate, have been demonstrated to improve ultraviolet stability [25]. Similarly, plant proteins and serum albumin have been shown to effectively complex with apple polyphenols and catechin, respectively, via non-covalent forces [14,26]. These representative examples, encompassing both covalent and non-covalent interaction types, are systematically summarized in Table 1. With regard to quantitative performance, sodium caseinate nanoparticles—which contain abundant proline residues capable of binding polyphenols—have been shown to increase the aqueous solubility of quercetin by 3034-fold and to significantly enhance the bioaccessibility of caffeic acid to 57.13% [27,28]. Whey protein isolates also serve as effective carriers for lipophilic polyphenols [29]. The reported functional outcomes primarily reflect enhanced bioaccessibility.

### 2.2. Polysaccharide–Polyphenol Interactions

Polyphenols are characterized by low aqueous solubility and poor chemical stability, which limits their full functional efficacy in the body. Consequently, a growing number of studies have focused on enhancing the water solubility and stability of polyphenols through polysaccharide-based encapsulation. The incorporation of polysaccharide chains into polyphenol–polysaccharide complexes can increase the hydrophilicity of the hydroxyl groups, thereby contributing to improved overall molecular solubility [30]. The water solubility and stability of quercetin, resveratrol, ferulic acid, and other polyphenolic compounds have been significantly improved owing to the introduction of polysaccharide substances, thereby enhancing their hydrophilicity [31,32]. Polyphenols and polysaccharides are primarily associated via both covalent and non-covalent interactions [33,34]. The main non-covalent binding forces between polyphenols and polysaccharides are hydrogen bonds and ionic and hydrophobic interactions [35]. The abundant hydroxyl (-OH), carboxyl (-COOH), and amino (-NH_2_) groups on polysaccharides provide multiple sites for hydrogen bonding and electrostatic interactions with the phenolic hydroxyl groups of polyphenols [30,33]. For instance, the amino groups of chitosan can be protonated to -NH_3_^+^ under acidic conditions, thereby facilitating electrostatic attraction with the negatively charged phenolate groups of polyphenols [15]. The covalent binding forces between polyphenols and polysaccharides mainly include oxidative bonding and free radical grafting [36]. These covalent interactions can occur spontaneously or be catalyzed by enzymes, acids, bases, or metal ions and can also be induced by free radicals to form polymeric structures [37,38]. Accumulating evidence has demonstrated that the interactions between polyphenols and polysaccharides predominantly involve the modification of functional groups, leading to structural alterations that enhance the stability of polyphenolic compounds, improve their biological activity and bioavailability, and expand their applicability in the biomedical, functional food, and chemical engineering sectors [34,39,40]. Compared to their monomer components, polyphenol–polysaccharide aggregates exhibit significantly enhanced antioxidant, antibacterial, and rheological properties. For example, standardized free radical quenching assays and electron paramagnetic resonance spectroscopy have confirmed that chitosan–sulfite copolymers significantly improved the scavenging capacity against reactive oxygen species [41]. Such polysaccharide–polyphenol polymers can serve as hydrogel additives for wound-healing applications. In particular, the prepared polyphenol–chitosan complex exhibits higher solubility, enhanced antioxidant activity, and superior rheological behavior, rendering it an ideal candidate for advanced wound-dressing materials [42]. Several studies have further explored the use of pectin and chitosan as raw materials to fabricate distinct nanocarriers for polyphenol delivery, thereby improving their stability and bioavailability [30]. By employing an electrostatic self-assembly method with chitosan and pectin to construct nanocarriers for blueberry anthocyanins, it was demonstrated that these nanocarriers effectively protect anthocyanins from degradation induced by oxidative reactions, thermal stress, and ultraviolet irradiation [30]. Moreover, Caenorhabditis elegans treated with these nanocarriers exhibited extended lifespan, enhanced reproductive capacity, improved locomotory activity, and reduced autofluorescent lipofuscin accumulation, further substantiating that chitosan–pectin nanoparticles can function as delivery vehicles to improve the stability and bioavailability of anthocyanins [15]. It has also been reported that polysaccharides can associate with polyphenols, thereby attenuating their degradation in the small intestine and increasing their metabolite levels in the colon, which consequently improves the bioavailability of polyphenols [43]. Furthermore, the encapsulation of polyphenols by polysaccharides can effectively shield them from adverse environmental factors. For example, curcumin encapsulated by gum nanoparticles was investigated for its stability in saline food systems; the results indicated that the curcumin content remained above 80% under high-concentration sodium chloride conditions, further confirming that gum nanoparticles can enhance the utilization efficiency of curcumin in high-salt food matrices [44].

### 2.3. Lipid–Polyphenol Interactions

Polyphenolic compounds suffer from low bioavailability and limited applicability, primarily owing to their poor solubility in both aqueous and lipophilic media, as well as their bulky polycyclic structures. These intrinsic physicochemical properties impede their absorption via non-active or passive diffusion pathways [45]. To address these limitations, a variety of strategies have been proposed, including the formulation of emulsions, liposomes, and nanoparticles [46,47]. Among these, phospholipid complexes have garnered attention as promising therapeutic agents. Phospholipids are amphiphilic molecules comprising both hydrophobic and hydrophilic domains, and their unique interfacial properties and favorable biocompatibility have rendered them highly attractive as pharmaceutical excipients or delivery carriers [48]. Phospholipids exhibit an innate affinity for polyphenols and are capable of forming non-covalent complexes, thereby enhancing the solubility and bioavailability of the latter. For instance, short-chain alkyl gallate–phospholipid complexes have been synthesized via ethanol evaporation, and these complexes have been shown to prolong the systemic retention time of short-chain alkyl gallates [49]. Similarly, phospholipid complexes have been reported to improve the bioavailability of resveratrol by inhibiting its glucuronidation and increasing its aqueous solubility [50]. With respect to quantitative delivery performance, lipid-based nanocarriers have also been extensively utilized for the delivery of polyphenolic compounds. For example, one study reported that the oral bioavailability of quercetin encapsulated in solid lipid nanoparticles was significantly greater than that of free quercetin. Nevertheless, it should be emphasized that this finding was obtained in a rodent model, and the quantitative extrapolation of these results to human subjects requires further validation, as interspecies differences in gastrointestinal physiology and metabolic enzyme profiles may influence the actual absorption efficiency [51]. Furthermore, Zhou et al. demonstrated that the gastrointestinal stability of lipophilic polyphenols (curcumin, resveratrol, and quercetin) in emulsion systems is highly dependent on their oil–water partitioning behavior, with higher oil-phase retention correlating with improved bioaccessibility [52]. More generally, while numerous studies report enhanced bioavailability of polyphenols via nanocarriers, most of these findings are derived from in vitro digestion models or animal studies. Accordingly, caution should be exercised when extrapolating these quantitative results to human applications, and further clinical investigations are warranted.

## 3. The Formation Mechanism and Influencing Factors of Polyphenol Nanoparticles

### 3.1. Preparation of Polyphenol Nanoparticles

Nanodelivery systems based on proteins, lipids, and polysaccharides have been developed for the targeted delivery of bioactive compounds [53]. These systems are designed to improve aqueous solubility, chemical stability, biological activity, and gastrointestinal absorption [54,55]. A range of preparation techniques have been employed, including phosphorylation, ultrasound technology, high-pressure homogenization, antisolvent precipitation, and pH-driven methods (Table 2). These techniques can be broadly classified into two general approaches for nanostructure fabrication. The top-down approach aims to reduce the size of structural materials to the nanoscale using precise tools or equipment and by applying external destructive mechanical forces. Common methods include thermal decomposition, ultrasonication and homogenizationFor example, high-pressure homogenization has been utilized to prepare soy protein isolate–resveratrol nanoparticles [54]. In contrast, the bottom-up method involves the use of self-organization/self-assembly of molecules and the formation of nanoparticles through nanoscale sedimentation. Representative examples include antisolvent precipitation for quercetin in corn protein/shellac/agar matrices [55], pH-driven assembly for curcumin in corn protein/shellac/phytic acid complexes [56], electrostatic deposition combined with antisolvent precipitation for anthocyanin in corn protein/polysaccharide carriers [57] and antisolvent precipitation for ferulic acid in quinoa protein [58]. Additionally, phosphorylation combined with ultrasound has been employed to prepare sunflower seed protein–quercetin nanoparticles [53], and cold plasma treatment of corn protein has been applied to encapsulate curcumin. Compared with untreated zein nanoparticles (encapsulation efficiency: 52.83%), the cold plasma-treated zein nanoparticles (85 W, 2 min) exhibited a substantially higher encapsulation efficiency of 76.31%, corresponding to a relative improvement of approximately 44.4%. In addition to improved encapsulation efficiency, the cold plasma-treated zein nanoparticles also displayed a smaller particle size (100.03 nm vs. 145.33 nm) and a higher ζ-potential (41.07 mV vs. 28.94 mV), indicating enhanced colloidal stability [59]. Additionally, ultrasound processing has been applied to soy protein isolate for the co-delivery of catechin and curcumin [60]. These fabrication strategies are broadly classified into top-down and bottom-up approaches [61,62]. Regardless of the method, strict control of compound ratio, concentration, temperature, and pH is required for optimal particle formation [63].

### 3.2. Factors Affecting the Stability of Polyphenol Nanoparticles

**Figure 2:**
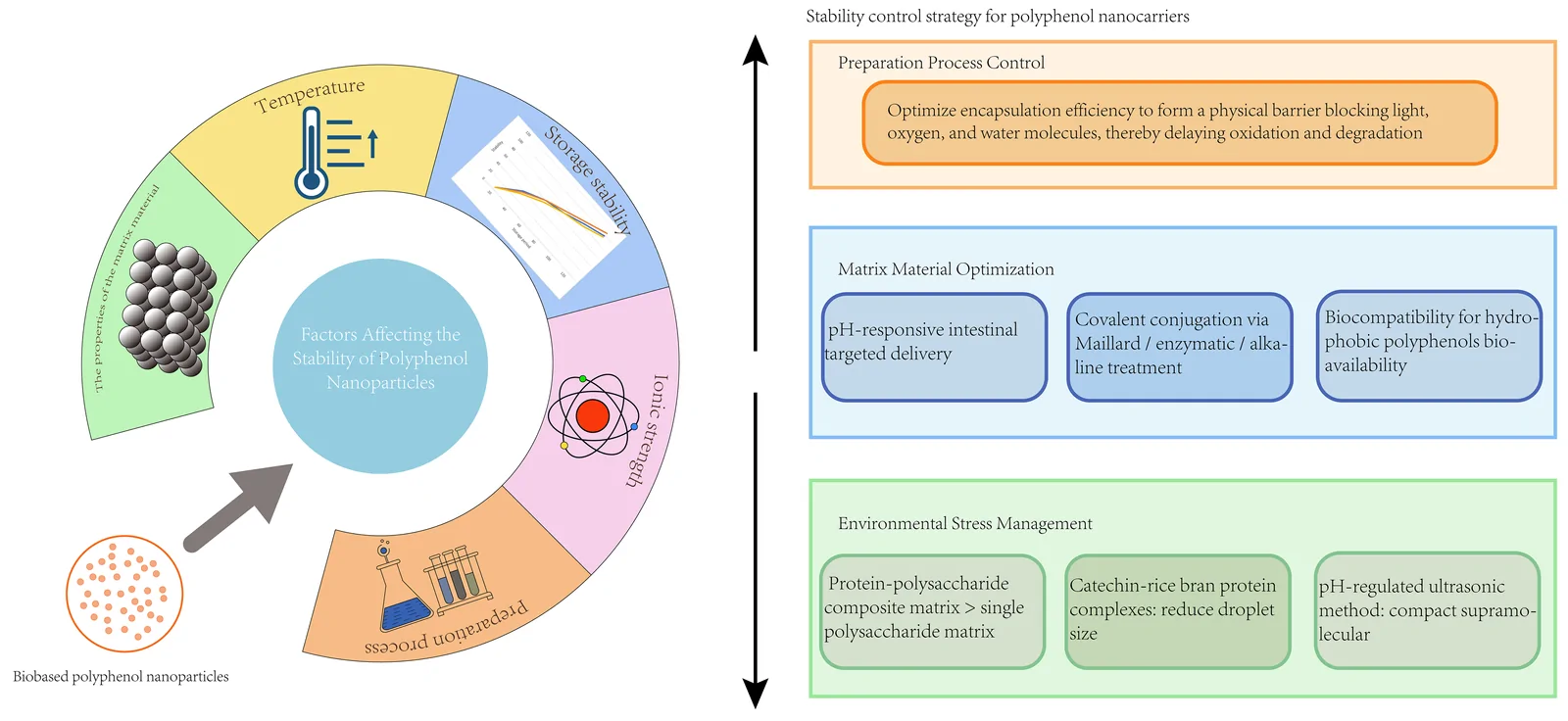
The multi-scale factors influencing the formation and storage stability of polyphenol nanoparticles and the control measures. Data sources: [66,67,68,69,70,71,72,73].

Owing to the poor stability of polyphenols under gastrointestinal conditions, the integrity of their molecular structure is difficult to preserve, which not only restricts their practical applications but also substantially compromises the efficacy of target tissues in executing their biological functions [64]. Consequently, recent studies have suggested that nanoencapsulation may represent an effective strategy to protect polyphenolic compounds from adverse environmental factors, thereby ensuring their stability and facilitating the broader implementation of this technology [65]. However, the formation and stability of polyphenol nanoparticles are influenced by multiple factors, which are schematically summarized in Figure 2 and discussed in the following subsections.

#### 3.2.1. Influencing Factors in the Process of Preparing Polyphenol Nanoparticles

Several critical parameters are involved in the preparation of polyphenol nanoparticles, which can influence both the efficiency and effectiveness of the fabrication process, as well as the stability of the resulting nanoparticles [74]. Particle size encompasses two interrelated aspects: dimensional scale and specific surface area. Smaller particles possess a larger specific surface area, which can enhance solubility and dispersion stability, thereby promoting faster absorption and ultimately improving bioavailability. However, smaller particles also exhibit higher surface energy and are more susceptible to aggregation, which may compromise their structural stability. Furthermore, very small particles are subject to rapid clearance from the body, thereby shortening their systemic retention time [66]. With regard to encapsulation efficiency, a high encapsulation efficiency not only serves as a critical indicator of preparation performance but also directly affects the storage stability of the final product. Unencapsulated polyphenols are more susceptible to degradation when exposed to light and oxygen, whereas effective encapsulation provides a physical barrier that retards the detrimental effects of external factors [67]. In the context of polyphenol nanoparticles, the carrier materials function as a protective shield for polyphenols, impeding the penetration of light, oxygen, water molecules, and other external agents, thereby delaying oxidative degradation and improving overall stability [66,67].

#### 3.2.2. The Influence of Matrix Material Properties on Polyphenol Nanoparticles

The physicochemical properties of matrix materials critically determine the stability and performance of polyphenol nanocarriers. As detailed in Section 2.1, Section 2.2 and Section 2.3, biopolymer matrices—including proteins, polysaccharides, and lipids—each offer distinct advantages with respect to encapsulation capacity, binding affinity, and controlled release behavior. Beyond these well-established attributes, each matrix type also possesses inherent limitations that must be taken into consideration during carrier design: proteins are sensitive to pH and thermal stress, which may induce denaturation and functional loss under certain processing conditions; polysaccharides, although conferring pH-responsive adhesion, may exhibit limited encapsulation capacity for highly hydrophobic polyphenols; and lipids, despite their excellent bioavailability-enhancing properties, are susceptible to oxidative degradation over prolonged storage [68,69,75,76]. Furthermore, polysaccharides can function as prebiotics, synergizing with polyphenols during gut microbiota fermentation to produce short-chain fatty acid metabolites, an advantage not shared by protein or lipid carriers [77]. To overcome the limitations of single-component systems, protein–polysaccharide complexes have been developed through covalent conjugation via Maillard reaction, enzyme catalysis, or alkaline treatment, yielding carriers with improved emulsifying properties, thermal stability, and antioxidant activity [70]. In therapeutic contexts, lipid-based nanocarriers have demonstrated additional benefits beyond bioavailability enhancement, such as improving diabetes treatment by facilitating glucose uptake and reducing oxidative stress [76]. Collectively, the selection of an appropriate matrix material—or combination of materials—should be guided by the specific application requirements, with composite systems often providing synergistic benefits that overcome the inherent constraints of single-component carriers.

#### 3.2.3. The Influence of Food Processing and Environmental Conditions on Polyphenol Nanocarrier Stability

In food processing applications, polyphenol nanocarriers are exposed to various environmental stressors, including storage duration, temperature fluctuations, and changes in ionic strength. It is important to distinguish between two related but structurally different colloidal systems discussed in the literature: (i) polyphenol nanoparticles, which are solid or semi-solid particulate carriers (10–1000 nm) composed of proteins, polysaccharides, or lipids; and (ii) polyphenol-loaded nanoemulsions, which are submicron liquid droplets dispersed in an immiscible continuous phase. Although these systems differ in architectural composition, their stability during processing and storage is governed by common environmental parameters, as summarized below.

The stability of polyphenol nanocarriers during food processing and storage is influenced by three major environmental parameters: storage duration, thermal treatment, and ionic strength. For nanoemulsion systems, droplet size increases over time due to flocculation and coalescence, whereas solid nanoparticles fabricated from protein-polysaccharide composites exhibit superior stability owing to enhanced interfacial and steric stabilization [71,78]. Thermal stability is improved by polyphenol incorporation, as demonstrated by flaxseed polyphenols in nanoemulsions and catechin–rice bran protein complexes, which reduce droplet aggregation and improve heat tolerance [71,72]. Ionic strength promotes particle aggregation through the weakening of electrostatic interactions, a challenge that can be mitigated by ultrasonic treatment or pH-regulated assembly to form more compact supramolecular structures [73,79,80]. The mechanisms underlying pH-responsive stability and controlled release are discussed in detail in Section 4.3.1.

The factors discussed above—including the inherent properties of polyphenols, the nature of the matrix materials, processing and storage conditions, as well as environmental parameters such as temperature, pH, and ionic strength—are systematically summarized in Figure 2. Understanding these influencing factors is crucial for the effective preservation of bioactive compounds and the improvement of taste and flavor characteristics in food applications.

## 4. Formation, Protection, and Sustained Release Mechanism of Polyphenol Nanoparticles

**Figure 3:**
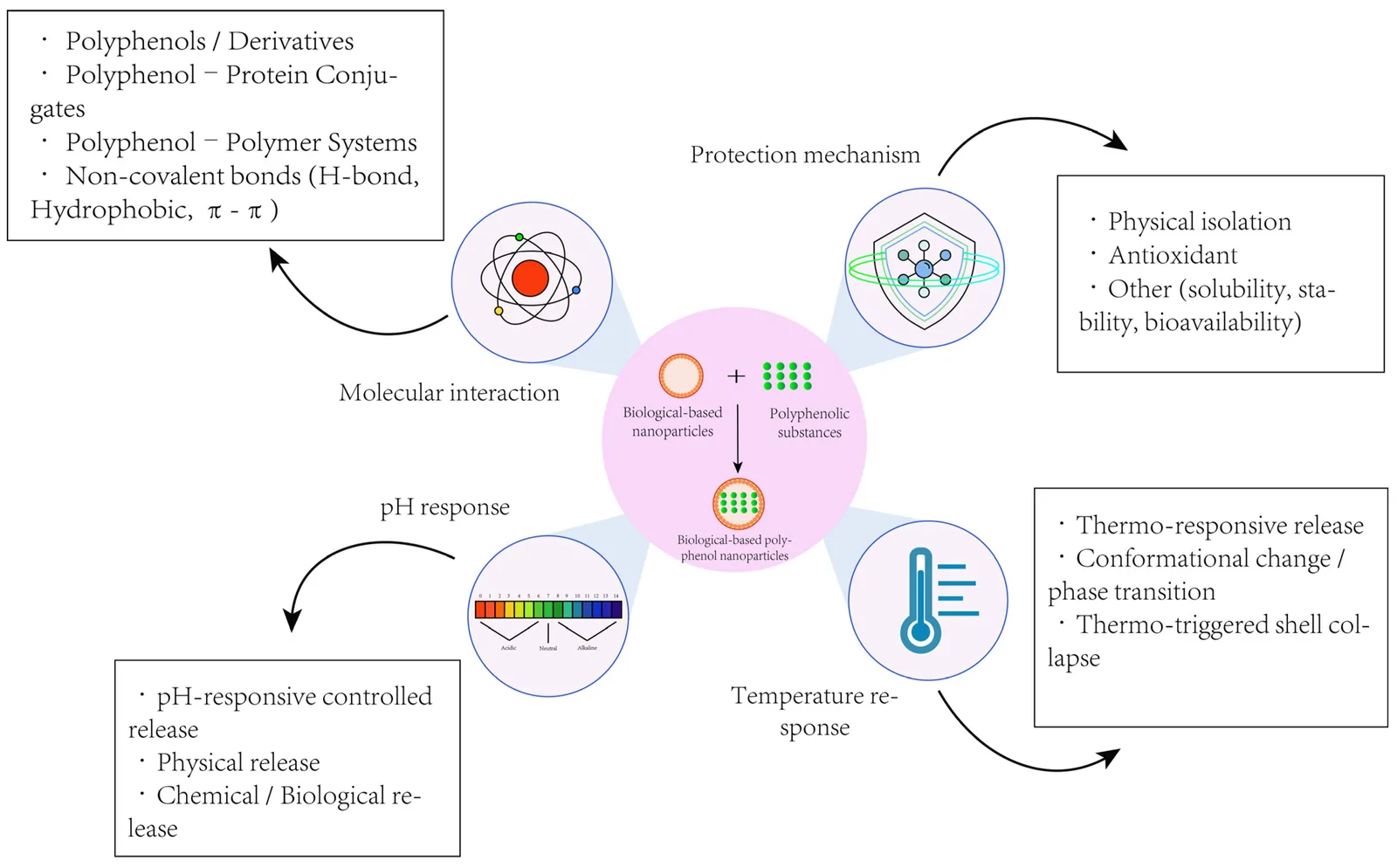
Integrated mechanism of polyphenol nanoparticles: formation, protection, and stimuli-responsive release.

Nanoencapsulation technology stabilizes these compounds at the physicochemical level, promotes their absorption and utilization in intact form, and enhances their bioavailability [81,82,83,84]. The functional efficacy of polyphenol nanoparticles arises from a sequential three-step process: first, dynamic self-assembly creates stable nanocarriers via non-covalent interactions; second, the matrix confers multi-dimensional protection against oxidation, pH extremes, and sensory defects; third, stimuli-responsive structures enable controlled release at target sites via pH or temperature triggers. The molecular interactions, environmental responsiveness (pH and temperature), and protection mechanisms underlying these systems are summarized in Figure 3.

### 4.1. Formation Mechanism and Interaction Between Polyphenol Nanoparticle Molecules

Polyphenolic compounds, as naturally derived bioactive molecules, not only exhibit therapeutic functions (e.g., antioxidant and anti-inflammatory effects) but also demonstrate robust non-covalent assembly capabilities mediated by their abundant phenolic hydroxyl and catechol groups [85]. These compounds can self-assemble into pH/enzyme/reduction–oxidation-responsive intelligent nanocarriers through weak intermolecular interactions (such as hydrogen bonds, hydrophobic interactions, and metal–ligand coordination) [86,87]. The polyphenolic self-assembly system offers three major advantages: (I) dynamic reversibility, which enables spatiotemporal control of drug release via pH, enzymatic, or redox reaction mechanisms; (II) functional integration, allowing the system to serve dual roles as both a therapeutic agent and a delivery carrier; and (III) excellent biocompatibility. Collectively, these synergistic characteristics have established a new paradigm for the development of efficient and safe drug delivery platforms [88].

The self-assembly of polyphenols refers to the spontaneous organization of molecules into ordered structures via non-covalent interactions. This process can occur either among polyphenols and their derivatives or between polyphenols and other molecules (including metals, proteins, and nucleic acids) [88]. The self-assembly interactions between polyphenols mainly rely on hydrogen bonding. In natural polyphenol complexes, the triad system of ellagic acid (EA), gallic acid (GA), and catechin (C) achieves a hierarchical assembly through an inherent molecular structure. Specifically, EA and GA initially formed two-dimensional structures through π-π stacking and hydrogen bonding. C promotes the final assembly of the “core–shell” lamellar configuration through a three-dimensional hydrogen-bonding network and hydrophobic interactions [89].

In addition to self-assembly among polyphenols, these compounds can also interact with proteins via hydrogen bonds and hydrophobic interactions, as detailed in Section 2.1 [88,90]. Furthermore, polyphenols can interact with modified proteins. For instance, modified butyl glycoside ether (BGE) side chains have been synthesized to generate water-soluble modified silk fibroin–BGE (SF-BGE), in which the phenolic hydroxyl groups of tannic acid (TA) form multiple hydrogen-bonding interactions. In addition, a coordination complex between zinc oxide nanoparticles and the catechol groups of tannic acid has been synthesized, resulting in a nanocomposite with multiple crosslinking networks [91].

The interaction between polyphenols and polymers, including polyethylene glycol (PEG) and polyvinylpyrrolidone, primarily occurs through hydrogen bonding. These polymers typically interact with the phenolic hydroxyl and galloyl groups of polyphenols [88]. The interaction between polyphenols and polymers, including polyethylene glycol (PEG) and polyvinylpyrrolidone, primarily occurs through hydrogen bonding. These polymers typically interact with the phenolic hydroxyl and galloyl groups of polyphenols [92]. The galloyl and catechol groups of TA form pH-dependent nanoparticles with the hydroxyl groups of PEG through hydrogen bonds in an aqueous solution. Specifically, as the pH increased from 6.6 to 8.3, the number of nanoparticles decreased, which may be due to the deprotonation of the phenolic groups in TA during the pH change [93].

### 4.2. The Protective Mechanism of Nanoparticles on Polyphenols

Polyphenol nanoparticles can confer multi-dimensional environmental stress resistance to polyphenols, which effectively isolates them from various external stressors, thereby maintaining chemical stability and biological activity during the product’s shelf life. With respect to antioxidative protection, biogenic nanoparticles play a critical role in physical separation by significantly reducing the permeability of oxygen, free radicals, and catalytically active metal ions into the core region, thus effectively inhibiting oxidative degradation [94]. Regarding pH stability, biogenic nanoparticles provide a relatively independent microenvironment for the encapsulated polyphenols, buffering against extreme pH values in the external food matrix. This physical isolation effectively prevents the core substances from undergoing hydrolysis, ionization state alterations, or precipitation induced by drastic pH fluctuations, whether in acidic beverages or in certain neutral or slightly alkaline food systems [95].

Nanopackaging can effectively shield bioactive polyphenols from adverse environmental reactions, protect labile polyphenols against oxidative and other chemical degradation, mask undesirable tastes and odors, reduce volatilization, modify physicochemical properties, alter the release profile from the wall material to prolong their duration, overcome solubility limitations, achieve controlled release, and ultimately enhance their bioavailability [29]. In addition, certain nanoparticles possess intrinsic antioxidative properties. For instance, although green tea extract is rich in polyphenols, it can induce various irritant skin reactions; however, when encapsulated with chitosan, the resulting nanoparticles exhibit protective antioxidant activity by enhancing polyphenol retention and scavenging reactive oxygen species [96].

### 4.3. The Sustained Release Mechanism of Nanoparticles on Polyphenols

#### 4.3.1. PH Response

A clear pH gradient is typically encountered during food processing, storage, and gastrointestinal digestion; consequently, pH-responsive release strategies have been widely adopted. This approach involves the integration of bioactive compounds with pH-sensitive carriers to achieve controlled release [97]. The application of this technology in food science contributes to extended shelf life, improved sensory quality, and enhanced food safety and public health outcomes. For example, in food preservation, a gelatin film containing eugenol-loaded nanoparticles exhibited acid-responsive release characteristics and effectively prolonged the shelf life of food products without compromising their quality [98].

In pH-responsive drug delivery systems, the release of active substances is controlled by physical mass transfer processes, chemical triggers, and biological activation mechanisms [99]. Among these, the fundamental release mechanisms dominated by physical processes can be categorized into three types: (I) diffusion-induced release, in which the active agent diffuses through the microporous or macroporous structure of the polymer and is transported from the film surface to the food matrix; the initial release of certain hydrophobic components is primarily controlled by a diffusion-mediated mechanism [100]. (II) Swelling-induced release occurs when a polymer is placed in a compatible liquid medium, allowing the liquid to penetrate the polymer matrix and cause expansion. In the swollen state, the diffusion coefficient of the active agent increases, thereby accelerating its release rate. This type of release is frequently observed in moisture-sensitive packaging materials, such as protein- and polysaccharide-based films [100]. (III) Decomposition-induced release arises primarily from polymer degradation, cleavage, or deformation, which results from changes in the physicochemical properties of the polymer matrix [100].

Chemical triggering and biological activation release can be classified into three types: (I) protonation of ionizable groups regulates electrostatic interactions, leading to swelling or contraction of the polyelectrolytes, which in turn can accelerate or decelerate the release rates. Specifically, carboxyl groups (-COOH) are protonated under acidic conditions, promoting contraction, whereas under alkaline conditions they undergo deprotonation to form hydrophilic -COO^−^ groups, resulting in swelling and subsequent release. In contrast, amino groups (-NH_2_) are protonated to -NH_3_^+^ under acidic conditions, facilitating their release, while at higher pH values, deprotonation leads to contraction and restricted diffusion [101]. (II) pH-dependent antioxidant activation and release: many potent phenolic antioxidants (such as ferulic acid and quercetin) have free radical scavenging activity that is pH-dependent and controlled by the acid dissociation constant (pKa) of their phenolic hydroxyl groups. In acidic solutions, the molecular form predominates, whereas in alkaline media (pH > pKa), the anionic form is the main species [102]. (III) Free radical-induced degradation as a release-triggering mechanism: in this process, the packaging system is designed to detect and counteract free radicals produced during food spoilage [99]. When the packaging system is exposed to oxidation-promoting conditions (such as light, temperature, and metal ions), a free radical chain reaction is triggered in the food, generating lipid peroxide free radicals (LOO) [103]. These LOO· free radicals act as key chain carriers, propagating the cycle by seizing hydrogen from lipids, thereby forming lipid peroxides hydrogen (LOOH), which then decompose into volatile odor substances and more free radicals [99]. For example, under acidic to neutral conditions, the formation rate of hydroxyl free radicals (·OH) is typically higher than that in alkaline conditions. At higher pH values, hydrogen peroxide (H_2_O_2_) is more prone to decomposition, thereby reducing its availability for the production of reactive oxygen species. Additionally, the overall reaction kinetics are impeded at higher pH levels [104].

#### 4.3.2. Temperature Response

Polyphenol nanoparticles that release their cargo in response to temperature control are referred to as thermosensitive polymer nanoparticles. These nanosystems are composed of amphiphilic copolymers, in which the hydrophobic core serves as a reservoir for polyphenolic compounds, while the hydrophilic shell provides colloidal stability [105]. Their temperature sensitivity enables them to undergo conformational or phase transitions upon mild temperature increases, thereby allowing the release of encapsulated cargo at target sites. When considering thermosensitive polymer nanoparticles, two fundamental parameters must be defined: the lower critical solution temperature (LCST) and the upper critical solution temperature (UCST) [106]. The LCST is defined as the temperature below which the polymer is soluble and above which it becomes insoluble, whereas the UCST is defined as the temperature above which the polymer becomes soluble and phase separation occurs. These critical temperatures are of paramount importance for the design and application of thermosensitive polymer nanoparticles, as they govern the conditions under which polymer solubility transitions occur, thereby modulating their structural behavior [106]. When the ambient temperature exceeds the LCST, the thermosensitive shell undergoes collapse around the core, thereby triggering the release of the encapsulated polyphenols [106].

## 5. Application of Polyphenol Nanoparticles

**Figure 4:**
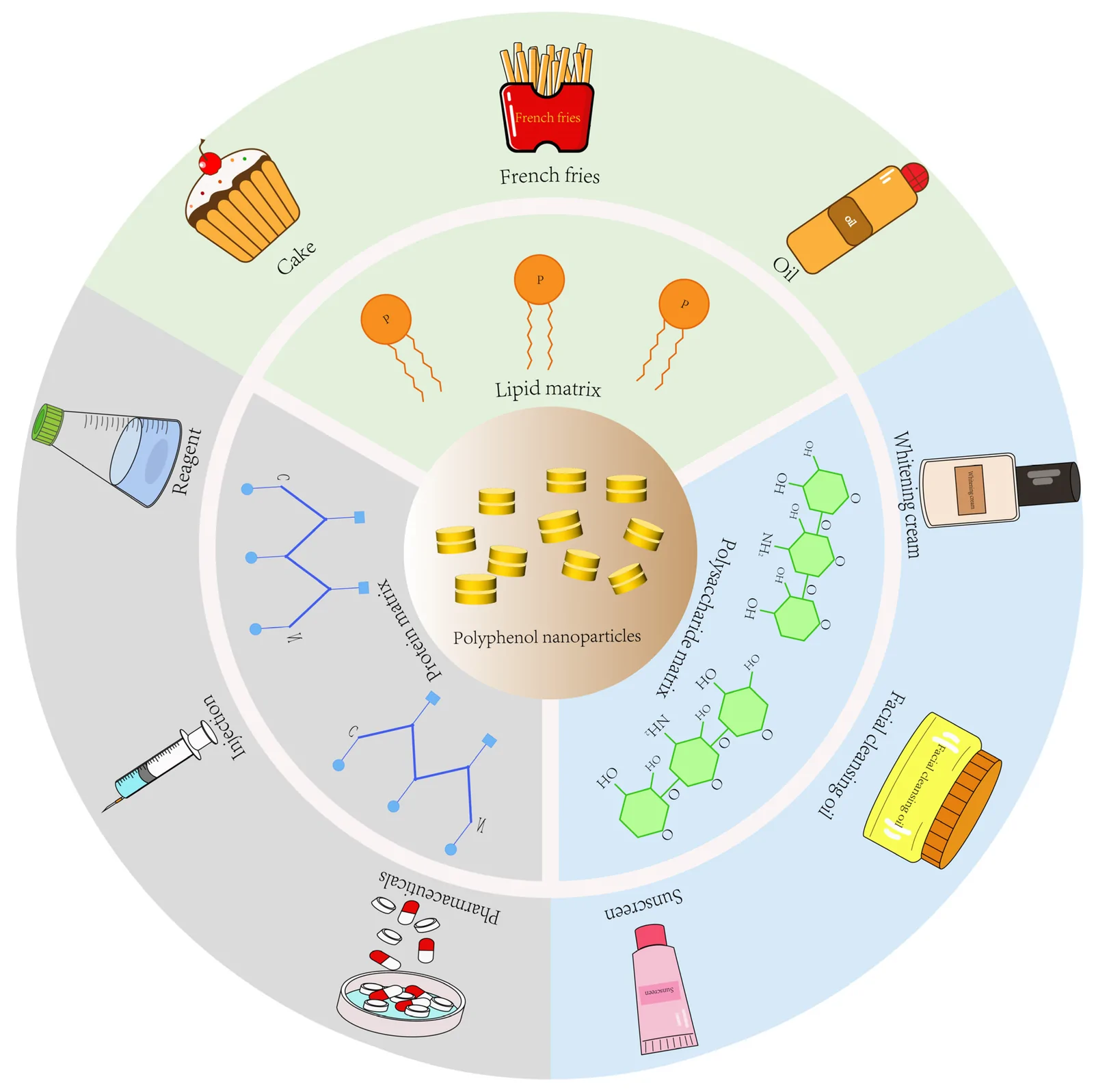
Radial schematic of bio-based polyphenol nanoparticle applications across food, cosmetic, and pharmaceutical sectors.

Bio-based polyphenol nanoparticles have demonstrated broad application potential across food, cosmetic, and pharmaceutical systems. The applications of these nanoparticles in these three sectors are illustrated in Figure 4. The following subsections provide detailed discussions of these applications in food, cosmetic, and pharmaceutical products.

### 5.1. Applications in Food

Edible oils, particularly those rich in unsaturated fatty acids, are highly susceptible to oxidative deterioration during processing and storage. This auto-oxidation process is initiated by metal-catalyzed free radical generation, followed by a chain reaction in which unsaturated fatty acids react with molecular oxygen to form hydroperoxides. These primary oxidation products subsequently decompose into secondary volatile compounds—including aldehydes, ketones, and short-chain acids—which are directly responsible for increased peroxide value (PV), p-anisidine value (p-AnV), and acid value (AV), as well as the development of off-flavors [107]. Concurrently, oils may undergo hydrolysis due to acids, bases, and high temperatures, generating free fatty acids (FFAs). Excessive FFAs can compromise oil quality, manifesting as unpleasant odors and poor taste. Notably, these oxidative and hydrolytic pathways are not independent; rather, they exert synergistic effects that accelerate oil deterioration during processing and storage [108].

The addition of antioxidants to scavenge free radicals is an effective strategy for preventing oil oxidation. Currently, the edible oil industry primarily relies on synthetic antioxidants for this purpose. However, accumulating evidence has raised concerns regarding their potential hepatotoxicity and endocrine-disrupting effects at certain dosage levels [109]. To mitigate the risks associated with synthetic antioxidants, regulatory agencies—including the U.S. FDA and the European Food Safety Authority—have imposed strict restrictions on their scope of use and maximum permitted levels in food products. Over the past few decades, natural antioxidants have garnered increasing attention owing to their high safety profile and potent antioxidant capacity. Polyphenols represent excellent natural antioxidants, exhibiting strong radical-scavenging activity and offering additional health benefits, such as cardiovascular protection, immune enhancement, and anti-aging and anticancer properties [110]. Nevertheless, the direct application of free polyphenols in oil systems faces substantial practical obstacles, including their hydrophilic nature—which limits solubility in lipophilic matrices—and their susceptibility to thermal degradation during processing. In food processing, nanoencapsulation technologies utilizing lipid-based or biopolymeric natural particles serve to protect labile bioactive compounds, such as polyphenols, from degradation induced by environmental factors including oxidation, light exposure, and pH fluctuations. These systems are designed to release their payloads in response to specific environmental or physiological stimuli, thereby enhancing the bioavailability and efficacy of encapsulated polyphenols [111]. In food systems, particularly in oils rich in unsaturated fatty acids, polyphenol nanoparticles function through three primary mechanisms: (i) free-radical scavenging to interrupt auto-oxidation chain reactions; (ii) physical barrier effects exerted by the nanoparticle matrix to retard polyphenol degradation; and (iii) controlled release under acidic or high-temperature conditions to sustain antioxidant activity during processing and storage. Collectively, these mechanisms contribute to reductions in peroxide value (PV), p-anisidine value (p-AnV), and acid value (AV), thereby extending shelf life. Despite these promising mechanisms, several critical challenges remain unresolved. First, the dispersion stability of polyphenol nanoparticles in high-fat environments is often inadequate, leading to aggregation and diminished efficacy. Second, the scalability of nanoencapsulation processes from laboratory to industrial production remains technically and economically constrained, with high energy costs and batch-to-batch variability serving as major bottlenecks. Third, the long-term safety and gastrointestinal fate of ingested nanoparticles have not been systematically evaluated, particularly with regard to their potential interactions with gut microbiota and intestinal epithelial barriers. Addressing these challenges will require interdisciplinary efforts that integrate materials science, food engineering, and toxicology to translate bench-scale innovations into practical food applications.

### 5.2. Applications in Cosmetic Products

Anti-aging, brightening, and whitening represent the most sought-after cosmetic benefits. Abnormal pigmentation is currently recognized as a primary contributor to skin aging and associated dermatological concerns [112]. Bioactive compounds, particularly polyphenolic compounds, have been extensively investigated in this context. Accumulating evidence indicates that these compounds can ameliorate both exogenous and endogenous aging processes, thereby preventing skin aging. Early studies reported that when resveratrol was encapsulated in solid lipid nanoparticles, its skin penetration efficiency was significantly enhanced, along with improved retention and cellular uptake of the encapsulated resveratrol. The encapsulated resveratrol exhibited enhanced antioxidant activity and stimulated collagen production while concurrently inhibiting tyrosinase activity and melanin synthesis, thus demonstrating notable skin-care effects [113]. Similarly, rutin and polyphenol-rich walnut oil extract have been encapsulated in solid lipid nanoparticles to prevent skin aging and pigmentation induced by solar ultraviolet radiation [114]. In cosmetic applications, polyphenol nanoparticles can address three major skin concerns: (i) anti-aging, by enhancing skin permeability and promoting collagen synthesis; (ii) brightening, by inhibiting tyrosinase activity and reducing melanin production; and (iii) photoprotection, by preventing pigmentation and oxidative damage elicited by ultraviolet exposure. Collectively, polyphenol nanoparticles represent a promising platform for cosmetic applications, as they enhance skin permeability, improve photostability, and enable targeted delivery of active compounds to the epidermal and dermal layers, thereby addressing key dermatological issues including aging, hyperpigmentation, and UV-induced damage.

### 5.3. Applications in Medicine

Nanoencapsulation can significantly enhance the bioavailability of encapsulated bioactive compounds, and adequate polyphenol doses are essential for the effective treatment of intestinal inflammation [75]. In the dextran sulfate sodium-induced mouse model of colitis, cellulose-based protein nanoparticles loaded with quercetin have emerged as an innovative therapeutic strategy for inflammatory bowel disease, leading to the upregulation of various pro-inflammatory cytokines. Chitosan-based nanoparticles containing quercetin have demonstrated comparable effects [75]. Relative to control colitis groups, treated mice exhibited significant downregulation of CD4 and CD8 gene expression, along with decreased pro-inflammatory cytokine levels and increased anti-inflammatory cytokine levels, indicating beneficial effects on the amelioration of colonic epithelial damage [115]. Furthermore, these polyphenol nanoparticles improved the species composition and relative abundance of the intestinal microbiota in mice with ulcerative colitis, suppressing the overgrowth of Bacteroides and Akkermansia genera and inhibiting the proliferation of pathogenic bacteria including Clostridium and Shigella, while promoting the growth of beneficial genera such as Lactobacillus, Propionibacterium, and Bifidobacterium [31]. In pharmaceutical applications, polyphenol nanoparticles exert therapeutic efficacy through two primary pathways: (i) anti-inflammatory regulation, achieved by downregulating pro-inflammatory cytokines and upregulating anti-inflammatory cytokines in colitis models; and (ii) microbiota modulation, accomplished by inhibiting the overgrowth of harmful genera (e.g., Clostridium and Shigella) while promoting beneficial bacteria (e.g., Lactobacillus, Propionibacterium, and Bifidobacterium). These dual actions collectively improve colonic epithelial integrity and restore intestinal homeostasis. In summary, polyphenol nanoparticles represent a versatile therapeutic platform for inflammatory bowel disease and other intestinal disorders, functioning through synergistic anti-inflammatory and microbiota-modulating mechanisms that collectively restore intestinal barrier integrity and immune homeostasis.

## 6. Conclusions and Future Prospects

This review systematically examines the preparation techniques, mechanisms of action, and broad applications of polyphenol nanoparticles across the food, pharmaceutical, and cosmetic industries. Nanoparticle encapsulation technology effectively addresses the inherent stability limitations of polyphenols by encapsulating them within carriers of 10–1000 nm, significantly enhancing their water solubility, chemical stability, and bioavailability. Different carrier materials offer distinct advantages: protein-based nanoparticles exhibit good emulsifying properties and biocompatibility; polysaccharide-based nanoparticles provide effective protection in the gastrointestinal tract due to their pH responsiveness; and lipid-based nanoparticles demonstrate higher encapsulation efficiency for lipophilic polyphenols. In food applications, particularly in oil systems, polyphenol nanoparticles improve stability by providing a physical barrier that delays polyphenol degradation and enabling controlled release under specific conditions to continuously scavenge free radicals, thereby reducing peroxide value (PV), p-anisidine value (p-AnV), and acid value (AV) and extending shelf life. In pharmaceutical applications, these nanoparticles enhance the bioavailability and targeted delivery of polyphenol-based therapeutics, offering improved treatment efficacy for various diseases. In cosmetic applications, they protect polyphenols from oxidation and photodegradation, enabling long-lasting antioxidant, anti-aging, and skin-protective effects in topical formulations.

In summary, polyphenol nanoparticle technology overcomes key application bottlenecks of polyphenols across multiple industries. Despite considerable progress, further efforts are needed to translate these advances into practical applications. Future research should prioritize comprehensive safety evaluations, including bioaccumulation, metabolic pathways, and long-term toxicity, as well as the expansion into personalized nutrition and precision medicine to meet the needs of diverse populations. With continued innovation in nanotechnology, polyphenol nanoparticles are expected to play an increasingly important role in the health food, pharmaceutical, and cosmetic sectors, contributing positively to human health and well-being.

## Figures and Tables

**Table 1:** Representative protein–polyphenol complexes: interaction mechanisms and functional outcomes.

Protein Type	Polyphenols	Reaction Type	Conclusion	References
Pea protein isolate	Curcumin	Glycosylation and covalent reactions	Pea protein isolate emulsification improvement; curcumin controlled release, and stability enhancement	[17]
Soy protein isolate	Catechin	Covalent reaction	Enhanced catechin bioaccessibility	[18]
Soy protein fiber	Curcumin, epigallocatechin gallate	Non-covalent (electrostatic interactions and hydrophobic interactions)	Ultraviolet stability improvement of this polyphenol type	[25]
Plant protein	Apple polyphenols	Non-covalent interaction	Replacement of animal proteins with plant proteins and enhanced apple polyphenol bioaccessibility	[26]
Serum albumin	Catechin	Non-covalent interaction	Effective catechin carrier function of serum albumin for in vitro application	[14]

**Table 2:** Representative bio-based polyphenol nanoparticles: preparation technologies, material systems, and targeted applications.

Materials	Polyphenols	Preparation Technology	Objective	References
Sunflower seed protein	Quercetin	Phosphorylation and ultrasonic technology	Seasoned lamb skewer preservation	[53]
Soy protein isolate	Resveratrol	High-pressure homogenization	The antibacterial properties of Staphylococcus aureus in beef	[54]
Corn protein, shellac, agar	Quercetin	Antisolvent method	Quercetin preservation with excellent stability, strong antioxidant capacity and controllable release	[55]
Corn protein, shellac and phytic acid	Curcumin	pH-driven method	Functional efficacy enhancement by curcumin and other hydrophobic bioactive compounds	[56]
Corn protein/polysaccharide	Anthocyanin	Antisolvent precipitation method and electrostatic deposition method	New strategies for anthocyanin packaging, stabilization and application expansion	[57]
Quinoa protein	Ferulic acid	Antisolvent precipitation method	Ferulic acid encapsulation optimization for quinoa protein-based polyphenol nanocarriers	[58]
Corn protein	Curcumin	Cold plasma technology	Cold plasma-assisted nanoparticle preparation for enhanced stability and encapsulation efficiency of nutrient delivery systems	[59]
Soy protein isolate	Catechins, curcumin	Ultrasound processing technology	Non-thermal technology for efficient delivery system fabrication and bioactive synergistic enhancement	[60]

## Data Availability

No new data were created or analyzed in this study. Data sharing is not applicable to this article.
